# A Night at the OPERA: A Conceptual Framework for an Integrated Distributed Sensor Network-Based System to Figure out Safety Protocols for Animals under Risk of Fire †

**DOI:** 10.3390/s20092538

**Published:** 2020-04-29

**Authors:** Oscar Tamburis, Francesco Giannino, Mauro D’Arco, Alessandro Tocchi, Christian Esposito, Giorgio Di Fiore, Nadia Piscopo, Luigi Esposito

**Affiliations:** 1 Department of Veterinary Medicine and Animal Productions, University of Naples Federico II, 80137 Naples, Italy; nadia.piscopo@unina.it (N.P.); luigespo@unina.it (L.E.); 2 Department of Agricultural Sciences, University of Naples Federico II, 80055 Portici (NA), Italy; giannino@unina.it; 3 Department of Electrical Engineering and Information Technology, University of Naples Federico II, 80125 Naples, Italy; darco@unina.it (M.D.); alessandro.tocchi@unina.it (A.T.); 4 Department of Computer Science, University of Salerno, 84084 Fisciano (SA), Italy; esposito@unisa.it; 5 CeRVEnE (Regional Veterinary referral Center for non-epidemic emergencies), 84031 Auletta (SA), Italy; giorgio.difiore@gmail.com

**Keywords:** fire risk, OPERA, animal safety, Distributed Sensor Network, simulation, disaster risk management

## Abstract

Large scale wildfire events that occurred around the world involved a massive loss of animal lives, with a consequent economic impact on agricultural holdings and damages to ecosystems. Preparing animals for a wildfire evacuation requires an extra level of planning, preparedness and coordination, which is missing in the current practice. This paper describes a conceptual framework of an ICT system implemented to support the activities of the Regional Veterinary referral Center for non-epidemic emergencies (CeRVEnE) in the Campania Region for the twofold objectives. On the one hand, it realizes the monitoring of the wooded areas under risk of fire in the so-called “Mount Vesuvius’ red zone”. On the other hand, it determines the **OP**timal **E**vacuation **R**oute for **A**nimals (**OPERA**) in case of fire, for each of the reported animal species living in the mentioned red zone. The main innovation of the proposed system lies in its software architecture that aims at integrating a Distributed Sensor Network (DSN), an ad-hoc software to generate timely simulations for fire risk modeling, and a GIS (Geographic Information System) for both the activities of web mapping and OPERA definition. This paper shows some effective preliminary results of the system implementation. The importance of the system mainly lies in its accordance with the so-called “Foresight approach” perspective, that provides models and tools to guarantee the prevention of systematic failure in disaster risk management, and becomes moreover critical in the case of Mount Vesuvius, which hosts a unique combination of both animal and anthropic elements within a delicate natural ecosystem.

## 1. Introduction

Any world’s complex system interacts with its environment, and this in turn features microsystems interacting with it: a large and delicate chain of interactions that, once broken down, not only affects the named system, but also the whole surrounding system in an irreversible manner (systematic failure) that leads to the loss of its function, thus causing a not readily recovering to a previous situation [1]. Under such a premise, providing a well-specific and shared definition for the term “disaster” is not an easy task, as it encompasses any kind of rapid-onset natural and man-made hazards, from disease spreading to avalanches, to railway accidents, just to name a few [2,3,4]. In particular, for what concerns fire-related disasters, the 2013 Annual Report of the United Nations office for disaster risk reduction stated that “[…] the devastating impact of forest fires on natural resources was neither quantified nor adequately took into account. Fires are harmful for a number of ecosystem services (whose loss is estimated at around 146–191 Bln dollars per year) including carbon storage, biodiversity support, protection of water sources, reduction of soil erosion, land degradation, and climate regulation” [5].

Accordingly, the whole set of actions itself to be defined, organized and eventually deployed to handle a disaster goes under many declinations, depending in this case on the specific aspects to be accounted for: the panorama spans therefore the concepts of disaster management (whose main focus is handling the events), risk management (which reviews trends with a concentration on the analysis of the risks and develops a certain response), and disaster risk management (which also has a look at trends and events, and proposes some actions) [6].

In this scenario, the CeRVEnE (Italian acronym for “Regional Veterinary referral Center for non-epidemic emergencies”) [7] was established in 2017 in the Italian Region of Campania, pursuing the objective of the Regional Government to improving and protecting people’s health through the timely management of both veterinary and non-veterinary epidemic emergencies related to animal health as well as to food safety. CeRVEnE’s mission is to analyze the consequences of disaster events, especially in social and economic terms, on territories, agricultural supply chains, and zootechnic systems, along with a particular emphasis on the Veterinary field: a structured approach to making decisions that aims at aligning with the main principles of the so-called “disaster relief supply chain quality management” (DRSCQM) system, as introduced by [8]. In accordance to this, among the initiatives of CeRVEnE, the FRAC Program (*Fire Risk Assessment in Campania Region*) was presented in 2018, as a project through which the Center intends to provide the Regional Government with a strategic tool able to: (i) gather and supply in a short amount of time all the necessary information to those professionals called to handle fire-related risks; (ii) have a large (both specialized and not specialized) public informed about the operational processes to get started after the fire damages in terms of safeguard and recovery of ecosystems (and related services), wild and domestic fauna, production supply chains; and (iii) figure out a standardized methodology for gathering data and supporting decisions.

The present work intends to describe the conceptual framework and to show some preliminary results of an ICT system to be implemented in order to support the activities presented in the FRAC Program. The aim is to enrich the line of inquiry into fire risk monitoring innovation by addressing a number of technical exigencies all-at-once, pursuing a twofold objective, that is on the one side to monitor the wooded areas under risk of fire in the so-called “Mount Vesuvius’ red zone”, and on the other side to determine the **OP**timal **E**vacuation **R**oute for **A**nimals (**OPERA** from now on) in case of fire, for each of the reported animal species living in the mentioned red zone. During a wildland fire, local animal rescue organizations’ and/or farms’ employees work with law enforcement and fire departments to rescue as many animals as they can. Such rescue operations are not well planned ahead, and typically depend on spontaneous volunteers in disaster response, despite the availability of government regulations such as the 2006 PETS Act in the US or the Directive 2010/63/EU within the European Union. Collaborative and ICT technologies in emergency response have a great impact and role, and there is quite a vast body of literature on this. Despite being used with proficiency in human rescue activities, sensor/cellular networks and decision support systems have not found the same application for animal rescue, yet. This study aims at filling this gap by proposing one of the first attempts for devising an ICT-enforced rescue system for companion, farm and wild animals.

The paper is organized as follows: after the Introduction, Section 2 describes the related research work conducted for disaster risk management and fire risk monitoring. The proposed ICT system is presented in three core sections. Specifically, Section 3 gives a model of the proposed ICT system analyzing the issues of creating a fire propagation map and defining a compatible evacuation plan; Section 4 surveys the infrastructures supporting the implementation of the ICT system; Section 5 focuses on the route optimization problem and the design of a system to grant its effective operation. The work also presents in Section 6 some preliminary results, including the analysis of a first-case scenario. Finally, Section 7 reports some concluding remarks.

## 2. Related Research Work

### 2.1. Disaster Risk Management

Disasters have always coexisted with civilizations. With technological advancements, development initiatives resulted in the creation of a lot of infrastructure and permanent assets, as well as in the thriving of the field of study concerning the management of disaster events, which includes the total sum of all activities, programs and measures that can be taken up before, during and after a disaster with the purpose to avoid it, reduce its impact or recover from its losses. The last decade of XX century was observed by the International Community as the “International Decade for natural disaster reduction“, and was dedicated to promoting solutions to reduce risks from natural hazards. The definition of fairly common vocabulary for the specific field was therefore attempted by many scholars: for instance, according to [9], hazard may be defined as “a dangerous condition or event, that threat or have the potential for causing injury to life or damage to property or the environment.” Hazards can be grouped into two broad categories, namely natural (e.g., cyclones, earthquakes, volcanic eruptions) and manmade (due to human negligence, such as chemical, industrial and nuclear accidents). Landslides, floods, drought, and fires are instead introduced as socio-natural hazards, since their causes are both natural and manmade. Vulnerability may be defined as “the extent to which a community, structure, services or geographic area is likely to be damaged or disrupted by the impact of particular hazard, on account of their nature, construction and proximity to hazardous terrains or a disaster prone area”. Vulnerabilities can be categorized into physical and socio-economic. Capacity can be defined as “resources, means and strengths which exist in households and communities and which enable them to cope with, withstand, prepare for, prevent, mitigate or quickly recover from a disaster”. People’s capacity can also be taken into account. Capacities could be classified into physical and socio-economic capacities. Risk is a “measure of the expected losses due to a hazard event occurring in a given area over a specific time period. Risk is a function of the probability of particular hazardous event and the losses it would cause.” The level of risk depends upon: (i) nature of the hazard; (ii) vulnerability of the elements which are affected; (iii) economic value of those elements. A community/locality is said to be at ‘risk’ when it is exposed to hazards and is likely to be adversely affected by its impact. 

In the wake of this, the authors in [6] pointed out some questions as to how to envision a picture of “future disasters”, such as: how reliable those visions are; whether the approaches applied to the future are on the right track; and whether there are any alternatives to improve current visions. To that end, the so-called “Foresight approach” provides models and tools to guarantee the prevention of systematic failure in disaster management [10]. As implied by [11,12], ‘the future’ is considered as emerging from the interaction of four components, namely: (i) events (those making people doubt the efficacy of thinking about the future at all); (ii) trends (a first type includes those being continuations of both the present and the past; a second type involves the cyclical patterns not being part of our own personal experience; a third type relates to emerging issues which are completely new); (iii) images (fears, hopes, beliefs, and concerns about the future); (iv) actions (based on the forecasts).

**Figure 1:**
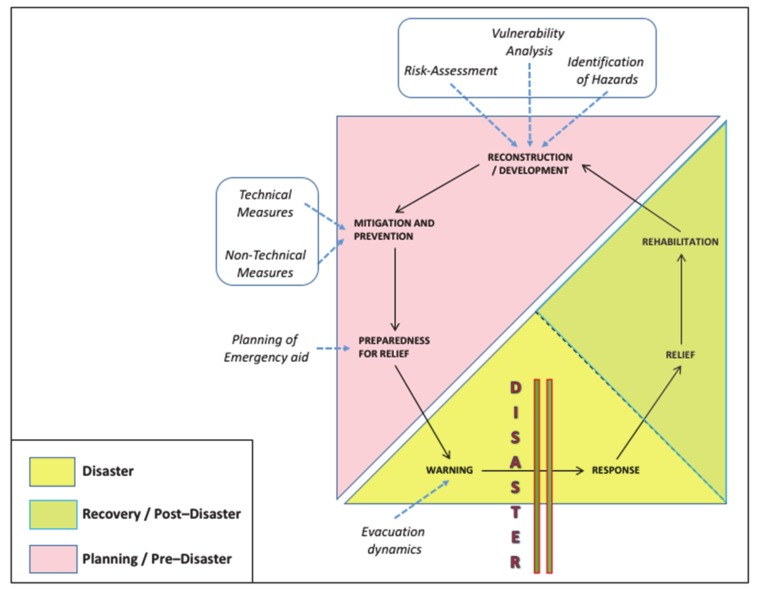
Disaster Management Cycle (elaborated from: Baird et al., 1975; Shorbi and Wan Hussin, 2015).

Critical importance is then assumed by the concept of “Disaster Management Cycle/DMC” (Figure 1), which shares isomorphic phases and associated concepts with the linear disaster phase [13,14]. Despite the alternative terminologies used in the literature (e.g., [2,15]), the Cycle aims at making clear that disaster and its management is not a series of events which start and stop with each disaster occurrence, but rather a continuum of interlinked activities. Other scholars [16] also derived the key disaster-related activities employing epidemiological methods, including rapid needs assessments, health surveillance, tracking systems, epidemiology investigations and studies, and registries.

The main phases of the DMC can be briefly described as follows [17]:

- *Disaster Impact*: point (in time and space) at which a disaster occurs. However, including it serves as a reminder that—in disaster management terms—impact can vary between different types of disaster.
- *Response*: encompasses all the measures usually taken immediately prior to and following disaster impact, the latter being the most frequent application of the concept. Such measures are mainly directed toward saving life and protecting property, and to dealing with the immediate disruption, damage, and other effects caused by the disaster.
- *Recovery*: means the process by which communities and the nation are assisted in returning to their proper level of functioning following a disaster. The recovery process can be very protracted, taking 5–10 years, or even more.
- *Development*: such a segment provides the link between disaster-related activities and national development. Its inclusion is intended to ensure that the results of disaster are effectively reflected in future policies in the interest of national progress.
- *Mitigation*: specific programs intended to reduce the effects of disaster on a nation or community.
- *Prevention*: means the set of actions designed to impede the occurrence of a disaster and/or prevent such an occurrence having harmful effects on communities or key installations.
- *Preparedness*: usually regarded as comprising measures which enable governments, organizations, communities, and individuals to respond rapidly and effectively to disaster situations.

As a dynamic entity, all phases of the DMC infer and involve action. This obviously requires a range of specialist facilities and systems, usually needed to cover things such as direction and coordination of disaster-related action, emergency operations center activities, as well as warning dynamics-related information management. Among the main contributions brought to the development of the DMC, worth mentioning are: the mentioned “Foresight Approach”, as its importance is being increasingly recognized by disaster managers and decision-makers for figuring out reliable long-term planning actions; the introduction of a social vulnerability index (SVI) that refers to the socioeconomic and demographic factors that affect the resilience of communities, after the original emphasis on infrastructure and technology [18]; the need to include animal issues into an overall emergency management strategy for a community [19].

### 2.2. Fire Risk Monitoring

The problem of early detection of fires in forest areas is widely recognized at both national and international level, as witnessed for instance by the European Forest Fire Information System (EFFIS), developed jointly by the European Commission (EC) services (Directorate General Environment and the Joint Research Centre) and the relevant fires services in the countries (forest fires and civil protection services) in response to the needs of European bodies such as the Monitoring and Information Centre of Civil Protection, the European Commission Services and the European Parliament [20]. Likewise, the Canadian wildland fire information system (CWFIS) is the national fire management information system, which elaborates daily information on fire weather, fire behavior potential and selected upper atmospheric conditions [21,22], while the Federal Land Assistance, Management and Enhancement (FLAME) Act of 2009 in the USA requires the Secretaries of Agriculture and Interior to develop a Cohesive Wildland Fire Management Strategy (CWFMS) and initiate a collaborative process between government and non-government agencies and devise solutions to wildland fire management issues [23]. A necessary look to Australia reveals a long story of forest fires (a long time before what occurred at the beginning of 2020), against which an as long tradition is standing of collection and elaboration of fire-mapping data derived from Moderate Resolution Imaging Spectroradiometer (MODIS) satellite imagery, and then, made available into the North Australia Fire Information (NAFI) website [24], along with the deployment of long-term and landscape-scale studies concerning the interactions between fire regimes and introduced livestock to pursue the conservation goal of population recovery for small mammals [25]. 

Forest fire is a sudden, strong and harmful natural disaster, which is why its quantitative analysis and modeling are important foci for many branches of research. Accordingly, different kinds of technologies have been developed and implemented for the measurement of the relevant parameters for early fire detection in risky areas. In particular, the use of GIS and remote sensing technology plays an important role in forest fire detection and prediction: on the one side, the implementation of hierarchical Wireless Sensor Networks (WSN) means the use of a number of sensing nodes that are capable of effectively gathering information from the surrounding environment and communicating with each other to send the measured data to a base station for further processing [26,27]; on the other side, Geographic Information Systems (GIS) can be used to combine different forest fire causing factors for attaining the forest fire risk zone map. GIS implementation allows the evaluation of a set of parameters that affect the fire, such as topography and vegetation, with other land use information including population, settlements, forest fire towers, fire stations, intervention places, the characteristics of the staff that will intervene, and transportation; this can make it possible for example to figure out the shortest way of intervention during the disaster, and/or the areas to be emptied [28,29]. 

With reference to EFFIS, this has been equipped with remote sensing techniques since 1998, while GPS tools are only used to determine fire perimeters. Although this methodology may be considered very precise for small fires, it introduces errors when mapping fires of large size. On the other hand, remote sensing is considered very reliable for mapping large fires, while less precise when mapping fires of small size, which may be omitted in the process of automatic classification of remote sensing imagery [30,31]. Moreover, many scholars developed general models to provide an assistant decision-making means for forest fire prevention systems, and thus, provide a scientific basis for disaster prevention, mitigation and assessment of post-disaster losses; it is e.g., the case of [32,33]. In other cases, the matter has been addressed for specific areas, such as Canada [34], India [35,36], Europe (e.g., [37]), or Mumbai [38]; in addition, [39] have studied the use of drones to map environments and survey them for items of interest such as forest fires, landslides or wild animals, thus gaining traction in various research communities.

## 3. ICT System Modeling

The software architecture presented in this paper aims at integrating a Distributed Sensor Network (DSN), an ad-hoc software to generate timely simulations for fire risk modeling, and a GIS (Geographic Information System) for both the activities of web mapping and OPERA definition. In particular, the latter step will be performed as an arborescence optimization problem to be solved, for instance, via label setting-correcting algorithms [40]. Such a framework is supposed to overcome some of the limitations emerged in the description of many of the current solutions adopted worldwide and descripted in the previous sections. Specifically, an effective integration of the single aspects proposed as components for our system is actually lacking, nor solutions capable of addressing all the issues of the DMC appear to be available. Moreover, the clear inclusion of animal issues within the larger dimension of the emergency management strategies seems not to be a “trendy topic”, whilst the inextricable connections between humans, animals, and surrounding environment emerges as one of the cornerstones of the One Health concept, which is inevitably called into account when dealing with the management of (not only fire-related) hazards [41,42]. 

**Figure 2:**
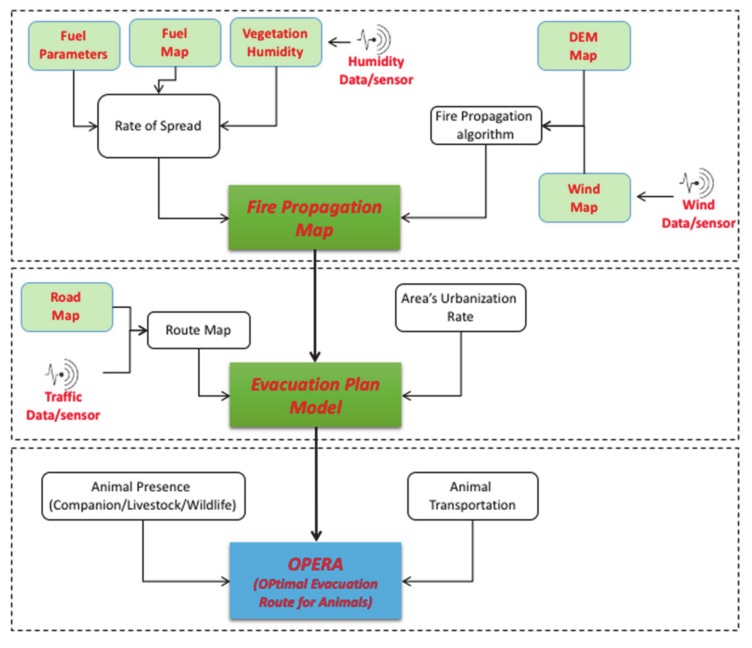
Architecture of the proposed conceptual framework.

Figure 2 depicts the architecture of the proposed framework [43]. At the beginning, a set of specific information is gathered to create a Fire Propagation Map, through which characteristics and dynamics of a fire episode can be analyzed and evaluated (represented in the figure in the first upper rectangle). Starting from this, an Evacuation Plan Model can be determined (the middle rectangle in the figure) by also adding anthropic-layered data related to both the urbanization rate and the road system development surrounding the area of interest, e.g., the Vesuvius in our study. For both steps, the deployment of specific sensors is required in order to collect the necessary mentioned situation- and context-aware information. Eventually, the model has to be enriched with the mapping of the animal presence (in terms of both typology and distribution) and the clear definition of type and size of vehicles requested to rescue the animal species involved in a fire episode. The determination of the OPERA for the involved rescuers (e.g., the beekeepers in our study) will be analyzed as a first-case scenario (in the lower rectangle of the figure), in order to the show the internal logic of the system as well as to provide some preliminary results as to its working dynamics.

### 3.1. Mapping of the Territories

**Figure 3:**
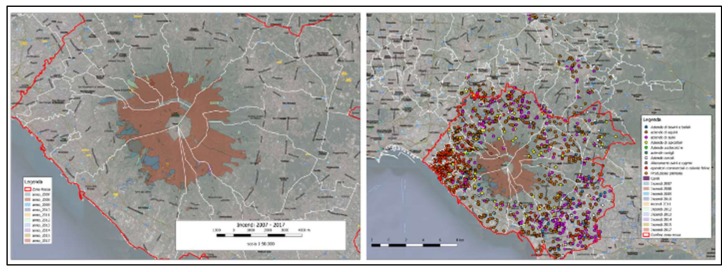
Map of the territories surrounding Mount Vesuvius, reporting fire risk areas (left) and farming activities classified according to the Fire Risk Assessment Matrix (right) (Source: FRAC program).

**Figure 4:**
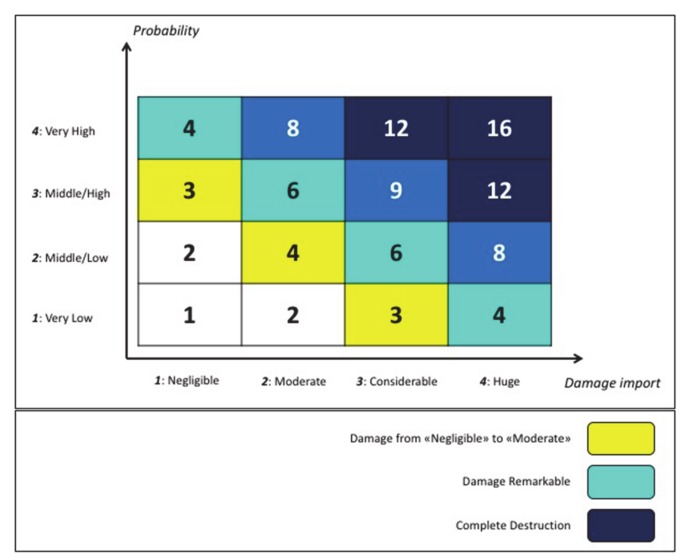
Fire Risk Assessment Matrix.

Satellite data represent the primary source of information for mapping areas under risk of fire. Figure 3 reports the main results of a first extended mapping of the territories surrounding Mount Vesuvius performed during the years 2018 and 2019 within the FRAC program. The purpose was twofold: (i) figure out a reliable map to classify the different fire risk areas, according to the Fire Risks Assessment Matrix (Figure 4) [44,45,46] (left side); (ii) detect and count all the farming activities concerning poultry, sheep and goats, cattle, bees, swine, and equines (right side).

### 3.2. TIGER Simulation Tool

With reference to Figure 2, the Fire Propagation Map is the output of the so-called TIGER simulation tool [47,48]. In particular, TIGER calculates a discrete time spread of the fire perimeter in a 2D landscape using two simple modules:

- the first allows calculating the rate of spread (ROS) maximum according to fuel type/vegetation distribution and vegetation humidity data;
- the second performs a geometric algorithm to calculate the perimeter evolution of the fire according to Digital Elevation Map (DEM) and wind data.

The effective ROS, derived by the calculation of the fire intensity / speed, is determined by the Equation (1) as follows:

(1) ROS_Effective=ROS_max·Wind_Effect·Slope_Effect

where: ROS_max (m/h) is the maximum ROS; Wind_Effect and Slope_Effect are the limiting factors of the wind and slope, respectively.

Moreover, each area covered by fire is described by a perimeter formed by nodes and segments. In the model, in each simulation step, the nodes move outward to form a new larger perimeter according to the ROS_effective parameter. Consequently, the model requires the following input data:

- DEM (digital elevation map), with a horizontal grid resolution of 20–40 m. The projection must be metric UTM WGS84;
- a fuel classification map based on the Anderson types [49];
- vegetation humidity data;
- observations and/or wind forecasts for the areas of interest (average speed and directions).

In the proposed system, the vegetation humidity and wind data will be provided from the implementation of an integrated sensor net distributed on the landscape.

## 4. ICT System Infrastructures 

An outline on the DSN duties are given as a foreword to a thorough analysis of the technical issues related to its terminal units, i.e., on-field devices. State-of-the-art technologies footing terminal units, network gateway, and structured data management are then reviewed, with the aim of figuring out background notes for an executive design of the DSN as a part of a much complex ICT system. Models, methods and software solutions that constitute the application level of the ICT system are discussed at the end of the section. (Anyway, although sensor networks based on wireless connections are becoming widespread because of their cheapness and easiness of installation, more general distributed sensor networks that allow coexistence of wireless and cable connections are here considered.) 

### 4.1. DSN Duties

DSNs have widely been investigated in the past, especially after the widespread deployment of wireless technologies, which have largely simplified and made cheap network installation and maintenance operations [26,27]. At present, one can say that the design of networks for a variety of applications is easily affordable. However, for severe working conditions, such as those related to the presence of fire or other kinds of harsh environments, the work of the designer becomes sensitive. The aimed DSN is in charge of detecting critical events, monitoring their dynamics, and, eventually, signaling early warnings. Additionally, it is capable of tracking people and animals’ activities in the area at risk, as well as informing about traffic jams along escape routes. The reliability of the aforementioned information is essential to the final goal of the overhead ICT system, devoted to real-time mapping risks and effectively implementing, on the need, the evacuation plan.

Due to the plurality of the targets demanded to the DSN, the network designer has to consider several technologies and arrange solutions to let them coexist; satisfying all specifications is otherwise unfeasible. Nonetheless, he has to obey the constraints for interfacing the network with the application level of the ICT system, where the algorithms for the control of high level operations, such as identification of optimal evacuation routes and most favorable flows scheduling, are executed.

### 4.2. DSN Technical Issues

The main technical issues at the design and deployment stage concern both the very purpose of the DSN and the requirements of it being a part of a multifaceted IT system.

Scrolling down the requirements of the DSN, scalability and upgradability stand as major ones. Scalability is intended as the possibility of integrating additional units in order to improve the basic network operation. Future upgradability is instead granted by conceiving the network as an open system with minimal constraints on interface requirements. In perspective, a relevant boost in the DSN’s performance is, in fact, expected from the utilization of Internet of Things (IoT) devices now under testing. Thanks to the inherent granularity of IoT systems, as well as to their connectivity and integrated adaptation schemes, the DSN will definitely accomplish improved robustness and reliability with the integration of compatible IoT units.

The electricity demand of the network units should be managed with efficient strategies, also considering the presence of pre-existing infrastructures. The typical sleep/deep-sleep operative modes can be exploited, which involve that all non-critical modules are disconnected from the power supply except during their short operative slots. Nonetheless, smart power modules complemented with solar, micro-wind, or piezoelectric energy harvesting systems can sustain the electricity supplies made of batteries or super-capacitors.

Concerning data communications aspects, a hybrid design approach, that merges both wireless and cable connectivity, can offer superior robustness and reliability; as for the electricity infrastructure, therefore, the presence of pre-existing cabling has to be considered. Hybrid solutions also provide additional degrees of freedom, thus offering a larger set of solutions for trading-off between number of terminal units, adopted communication means, complexity and costs of the network.

The availability of a network clock signal is necessary for synchronization purposes. To this end, gateways equipped with low-cost GPS receivers are opportune. The network can be complemented with an additional protected gateway, implementing the black-box concept, such that post-disaster analysis is made possible. The protection of the black-box gateway represents a sensitive aspect and has to be assured by physically placing, or constructing for it, (at least for the critical parts of its hardware) a safe sheltered site.

In order to face issues related to harsh operating conditions, like those experienced during forest fires, the designer has to assure sufficient operating time also to other selected critical equipment, considering for them the use of superior casing structures, like IP67 anti-fire packages. The design cannot jeopardize maintenance issues after the DSN deployment. In particular, the on-field units of a DSN represent hot entries in maintenance programs, since most of them claim for on-site operations, such as battery replacement, which is troublesome if the unit is installed in a harsh environment. To this end, new prognostic and diagnostic methods, based on Artificial Intelligence (AI) approaches, can offer relevant benefits, in terms of faults reduction, improved overhauls rapidity, and maintenance costs as a whole. An AI sub-system supporting the DSN maintenance program can be included at application level in the ICT system. 

Conclusively, the designer has the responsibility of finding the synthesis that best satisfies the mandatory requirements and grants compliance with standards that are applicable to network topology, specific hardware and software solutions, as well as to energy supply and ancillary energy harvesting systems. 

### 4.3. State-of-the-Art Technologies Footing DSN

**Figure 5:**
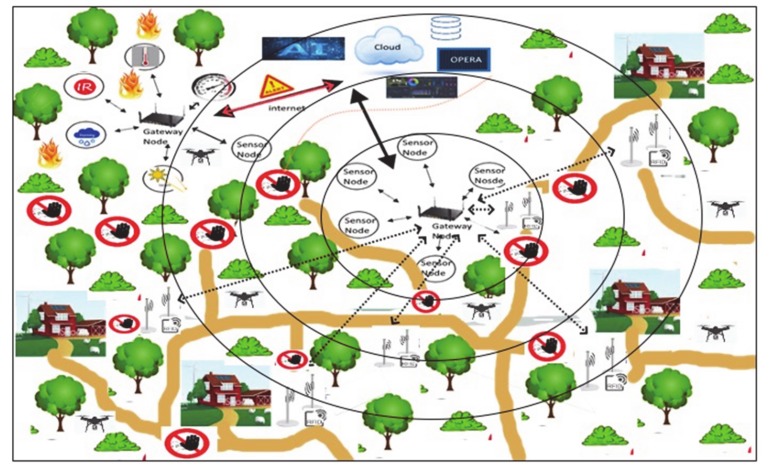
Schematic representation of a DSN for early detection and monitoring of fire in forest areas, eventually integrated with several facilities made available by IoT solutions.

**Figure 6:**
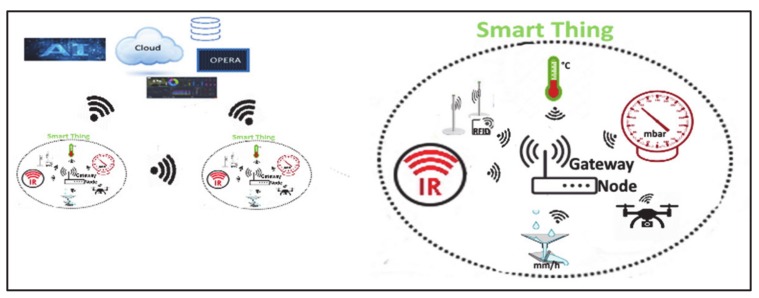
Smart devices with sensing and actuating capabilities and their inter-networking.

At the state-of-the-art, the innovative paradigm enabling IoT applications, sketched in Figure 5, seems to offer superior advantages in both design and implementation of a candidate DSN with respect to more traditional solutions. The DSN therefore has to be conceived as a network of smart devices, or smart things, with sensing and actuating capabilities, as sketched in Figure 6.

Smart devices mainly consist of sensors/actuators and gateway sections. Each sensor includes a smart power module, one or more probes, a processor, such as the one available in commercial boards hosting system-on-chips, and a communication module. The latter is often optionally included in the processor board; if not, it has to be connected as an external unit to obtain connectivity. Probes typically include an analog front-end and electronics for preprocessing the input. Most of them even provide analog to digital conversion, local data storage, and one or more standard digital interfaces. Smart detectors for smoke, relative humidity, temperature, atmosphere pressure, mono and dioxide carbide (CO, CO_2_) percentages, solar irradiance, rain percentage, infrared/ultraviolet radiation intensity, volumetric water content and suction in soil, including the aforementioned characteristics, are commercially available. More recently, unmanned aerial vehicles (UAV), also known as drones, have successfully been used as sensors/actuators, thus showing the possibility of overtaking the barrier of the static concept of sensor in a DSN. Drones offer dynamic configurability and widen DSN terminal units to new kinds of operations not possible with fixed units. Furthermore, DSN units can include radio-frequency identification (RFID) options, by hosting one or more tags, and allow the intervention of personnel at the network level with the use of a handheld reader device. The tags exchange short data packets with the reader by exploiting the back-scattering principle, namely by radiating back a modulated version of the received signal, where the modulation is used to convey information. The reader equipment is made up of a power module, a processing machine and an RF transponder; it extracts the information from the on-field unit and makes it readable to the technician.

More in detail, RFID tags can be classed into active, passive and semi-passive. Active RFID transponders are self-powered, whereas passive ones are powered with an external electromagnetic signal. For passive tags, the reader equipment transmits energy, which is temporarily stored as charge in an auxiliary capacitor complementing the tag. Further classification is based on the adopted frequency band: the low-frequency (LF) tags operate in the range 125 up to 135 kHz, the high-frequency (HF) ones use the band centered at 13.56 MHz, the ultra-high-frequency (UHF) ones use the bands centered at 433, 868 and 956 MHz; the very last RFIDs on the market can use the free microwave band centered at 2.4 GHz. UHF and microwave RFIDs need shorter antennas with respect to LF ones and allow reading operations at longer distances, up to 10 m. UHF frequency range is also well suited to tracking applications, where fixed readers interrogate tags associated to animals, for instance inserted in a collar, to identify them and extract further information, which can be useful during emergencies events.

Data transmitted by terminal units should match simple structures and be concise, so that traffic between terminal units and gateways can easily be upgraded to upcoming technologies and communication protocols. Concise data and low data rates cope with the low power requirements of long-range wide-area networks and related protocols. At present, LoRaWAN, a novel wide range distance communication protocol robust to noise and interference, and capable of offering a coverage radius up to 15 km, deserves particular attention since it can be implemented using ultra-low power electronic circuits. A variety of low-cost equipment recently presented on the market supports long-range data transmission and allows rapid prototyping network solutions. For instance, there are starter packs and evaluation boards offered by major manufacturers that combine powerful 32-bit microcontrollers with several different expansion boards. These can include several micro electro-mechanical sensors (MEMS) as inertial (accelerometer and gyroscope), pressure, humidity, and temperature sensors. The use of proprietary solutions that operate as serial long-range transmitters, where data are encapsulated in key-value proper structure and transmitted to the gateway, should be considered as a secondary spare.

### 4.4. State-of-the-Art Technologies Footing Network Gateway and Structured Data Management

Gateways collect data from the terminal units deployed on field and forward them to the core processor of the ICT system. A network can include several gateways, each one connected to a subset of the terminal units. More specifically, gateways have to preprocess and merge the atomic data produced by the terminal units into structured information, and transmit it to the ICT platform. They require much more hardware in terms of processing and memory resources than terminal units.

Commercial boards, such as BeagleBone and Raspberry-pi, represent viable choices. They are very cheap single-board computers based on a renowned operative system (Linux), which can be equipped with additional modules for signal conditioning, analog to digital conversion, and wired/wireless standard communication interfaces. By exploiting the local storage capabilities of gateways, copies of critical data can be temporarily saved, thus avoiding information loss in case of occasional connection failure.

Gateways can communicate with the overhead ICT platform by means of either standard mobile or Ethernet protocols or message queue telemetry transport (MQTT) protocol, a publisher/subscriber application protocol fancied by IoT developers. Data refresh can be programmed at a low rate, which is sufficient for regular monitoring. The network can be designed such that data refresh is automatically increased during emergencies, eventually by complementing LoRa technology with redundant solutions. These can rely on different technologies capable of assuring wider bandwidth even at the expense of increased power requirements and shorter range coverage, and a suitable dynamic switching strategy.

Merging atomic data to produce structured information involves a distributed and hierarchical database architecture. In this scenario, each gateway plays its own role by saving data of a set of terminal units, namely those directly connected to it, and manage a portion of the whole database assembled by the overhead ICT system. Additionally, a subset of primary gateways of the network can be equipped with additional memory resources to store clones of the local databases hosted by adjacent gateways; this assures more robustness of the network in terms of capability to keep safe the collected data. Redis or MongoDB databases represent ideal candidates. They are recent solutions featuring innovative storage and data retrieval mechanisms, which are different from the tabular approaches used in relational databases. In particular, Redis is an open source in-memory data store, that grants high availability and fast responsiveness. It can cope with many data structures, from simple ones as strings to more complex ones as hash tables and lists. Nonetheless, its built-in replication mechanism, implemented according to a master/slave model, mostly simplifies the production of exact copies of database instances. It provides automatic partitioning, which is a way to automatically split and distribute data across different memory units. It offers a mechanism of self-diagnostics to constantly check if master and replica instances are synchronized. 

Finally, the overhead ICT system can integrate cloud computing services that simplify storage and computational tasks, and produce more consistent, accurate, and useful information than atomic systems. Cloud services also represent efficient solutions for the implementation of artificial intelligence (AI)-based approaches, which are becoming important backings to solve very complex problems. 

## 5. Route Optimization and Effective Implementation

### 5.1. Optimization Model

Defining OPERA means setting up an arborescence optimization problem, that could be tackled via the implementation of a label setting-correcting algorithm. In order to model such a problem [50], let then G = G(N, E, φ) be an oriented graph, where:

- N = *nodes*: set of all the accessible road junctions in Mount Vesuvius’ red zone;
- E ⊆N x N  = *edges*: roads that connect two consecutive junctions/nodes. It can be stated that: $e_{k}=n_{k},\;n_{k+1}$;
- $\varphi :E\to \left[0,1\right]$ = *weight function* (normalized) that assigns to each edge a value of intensity of traffic flow (defined in the continuous interval from 0 to 1).

Let moreover be:

- $t\;\in [0,\;t^{*}[\;\subset [0,+\infty [$ a discrete time interval, where t* represents the final moment of the simulation;
- $n_{k,t_{i}}\in N$: junction occupied by the *k*-th token at the moment *t_i_*.

Under such premises, some conditions can be defined. In the first moment, for any interval [*t*_i_, *t*_i+1_], it is possible to identify at least one edge, as expressed in Equation (2):

(2) $$\forall t_{i},t_{i+1}\in [0,\;t^{*}[\;,\exists \;n_{k},\;n_{k+h}\in E,\;with\;n_{k},\;n_{k+h}\in N$$

For each moment *t*_i_, each junction/node could be/could not be interested by the fire spreading. This can be expressed by introducing a function ρ as reported in the Equations (3) and (4):

(3) $$\rho :N\;x\;[0,\;t^{*}[\to \left[0;1\right]$$

where:

(4) $$\rho =\left\{\begin{array}{c} 0,\;\;n_{k}\;reached\;by\;the\;fire\;at\;t_{i}\\ 1,\;\;n_{k}\;not\;reached\;by\;the\;fire\;at\;t_{i} \end{array}\right.$$

If the token is in *n_k_* at the moment *t_i_*, and from there many alternative routes are available, it can be assumed that there will be only one *n_k+1_* junction related to the edge *e_k_* with the least intensity of traffic flow. This is expressed in Equation (5) as:

(5) $$\forall n_{k,t_{i}}\in N\;x\;[0,\;t^{*}[\;,\exists !n_{k+1}\in N\;with\;e_{k}\in E:min\varphi \left(e_{k}\right)$$

A further condition to be added to the previous one is that assigning a value of intensity of traffic flow to an edge means as well evaluating the most likely average speed a vehicle will deploy to cover the edge itself [51]. To that end, Equation (6) describes the average speed evaluated for *n* vehicles in a given time period:

(6) $$S_{t}=\frac{1}{n}\cdot \overset{n}{\underset{i=1}{\sum }}S_{i}$$

where: *S_i_* = speed of the *n*-th vehicle. As a consequence, the average speed evaluated on a given distance is expressed by Equation (7) as:

(7) $$S_{S}=\frac{n}{\sum_{i=1}^{n}\frac{\Delta t_{i}}{L}}=\frac{1}{\frac{1}{n}\cdot \sum_{i=1}^{n}\frac{1}{S_{i}}}$$

Besides the speed average values, also necessary are: (i) the evaluation of the size and the number of the vehicles requested to transport the animals; (ii) *S*_85_ = the 85th percentile of the variable *S*, that is the speed only excessed by 15% of vehicles; and (iii) *V*_mod_ = most frequent cinematic interval. 

The complete model is presented as follows, with which the objective is to minimize the escape route for each of the reported animal species living in the Vesuvius’ red zone in case of fire, in order to the definition of effective safety protocols from CeRVEnE for the FRAC program.

(8) $$OPERA=min\overset{N}{\underset{k=1}{\sum }}\rho (n_{k,t_{i}})\neq 0$$

*s.t.* (2)–(7)

OPERA poses very similar characteristics with the category of *exact problems*, for which the corresponding optimization problems can be polynomially solvable [52]. What was here presented is the attempt to find out a timely heuristic to provide an acceptable solution to the problem, even considering the peculiar context of application, as the core for the realization of a distance analysis tool for the GIS module of the integrated system at study to be endowed with [53,54,55].

### 5.2. Design of a Real-Time Routing Tasks Helping System

Section 4 has surveyed duties, technical issues, and available technologies for DSNs, with the main intent of showing the large number of possibilities at disposal of the designer. Additionally, network gateways and related system logics have been illustrated, still adopting a very general point of view, aiming at delineating a framework useful to the designer for tailoring solutions that can cope with general requirements.

**Figure 7:**
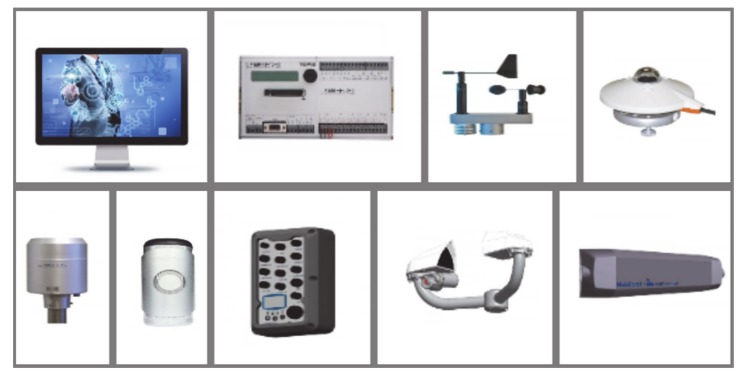
Image-list of compatible commercial products to set-up the system infrastructure. The system is capable of collecting the critical information needed to a real-time implementation of the route optimization strategy. On the top, from left to right: monitoring management and control software platform, meteorological station unit, wind speed relative humidity and atmospheric pressure sensors, solar irradiance sensor. On the bottom, from left to right: rain gauge, environmental sensor (CO, NO_2_, O_3_, PM10), multifunction local control unit, visibility sensing unit, microwave traffic counter and traffic classifier.

Hereinafter, a specific solution for the implementation of the optimal escaping plan, according to the route model discussed in Section 5.1, is presented. The solution includes products for traffic flows, meteorology and environmental measurements, and software packages that can be exploited by means of ubiquitous facilities, like tablets or smartphones. An image list of products, available on the market and compatible between each other, to set up the proposed solution is given in Figure 7.

Traffic flow measurements can be carried out by means of non-intrusive and low power sensors available on the market for counting and classifying vehicles as well as measuring speed parameters, as required by the considered model. These sensors are typically installed on the center of the monitored lane, and, thanks to microwave technology and time-of-flight measurements of the electromagnetic signal, are capable of detecting the vertical profile of the vehicles (useful to the classification purpose), the direction of travel, and the corresponding speed. They are also capable of detecting headway and gap, the time the lane is occupied, and stop-and-go or stationary events, which are typical marks of a traffic jam. They are complemented by a firmware-hosting algorithm to identify accidents, overtaking, open worksites, and to perform statistical analyses in order to estimate average speed and percentiles, like those required by the model in Section 5.1. The sensors can be deployed on a selected subset of roads to directly sample the traffic flows, whereas software approaches can be exploited to infer traffic data on secondary roads.

Meteorology data are available at a marginal cost in systems like the described one by deploying low-cost and low power ancillary sensors. These offer valuable help in real-time forecasting programs by presenting weather data such as atmospheric temperature, precipitation, wind knots and direction, and visual range. It is worth noticing that temperature and visual range parameters are highly correlated to fire events and smoke, and can be used as indirect detectors or as options contributing in redundancy at monitoring. Similarly, environmental sensors can be included in the system with a marginal impact on the cost. They allow measuring air quality parameters such as carbon monoxide (CO), nitrogen dioxide (NO_2_), ozone (O_3_) and particulate matter (PM10), which are highly correlated to traffic and industry operation, too.

All the considered sensors can be powered by the electrical network, or alternatively by photovoltaic panels, or even battery, and are designed for low power consumption and energy savings. Nonetheless, the designer of the system can rely on customer programs by major manufacturers, according to which the aforementioned products are sold as open solutions, which can be complemented with optional functions implemented on demand. 

Taking into account the specific application needs, traffic, environmental and meteorology sensors can be deployed in proximity to each other and connected to the same local control unit. The latter has to integrate standard interfaces for data transmission to remote collectors as well as on-site configuration and firmware updates. The local control units at present available on the market host one or more standard interfaces that can be selected among Ethernet, Wi-Fi, or 3G and 4G cellular networks, which are all capable of supporting even internet browsers. 

The hardware of the communication infrastructure and the processing capabilities of the local units can be fully exploited when stepping from the peripheral level to the central level, where data from all local units are gathered, processed and validated. The central level system is in charge of supporting the main actors during the hazardous event. To this end, the central mainframe has to offer a service that should be accessible by the subject in an easy and effective way, for instance by means of a mobile application (app) designed for tablets and smartphones (to be further explored in the next phase).

## 6. Preliminary Results

The first case scenario chosen to figure out the proposed framework focuses on beekeepers as the beehives’ transportation is in general less problematic in terms of general set up and execution.

**Figure 8:**
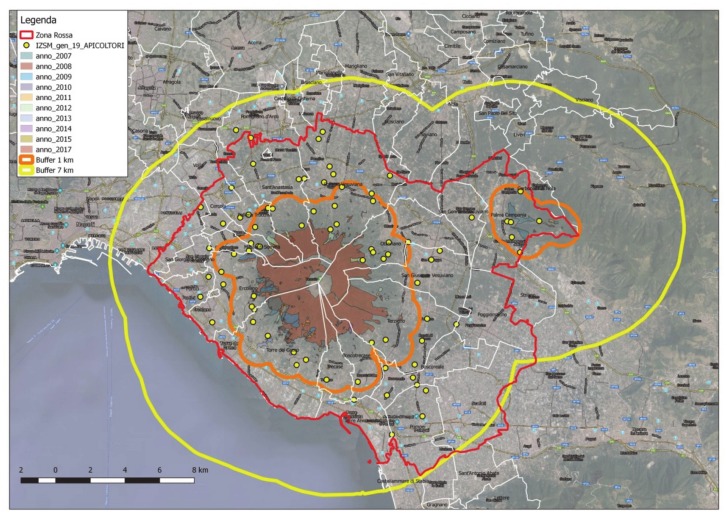
Buffer zones interested by direct (inner zone) and indirect (outer zone) effects of fires on beekeepers’ farms for the period 2007–2017.

The geospatial data provided from the South Italy Experimental Zoo-prophylactic Institute (Istituto Zooprofilattico Sperimentale del Mezzogiorno, IZSM) concerning the fires that occurred in the Mount Vesuvius surrounding area during the years 2007–2017, made it possible to localize all the beekeepers’ farms, other than collecting information as to the negative impact of the fires on their production dynamics. Since it is known that the maximum flight range of a bee swarm is of 7 km from the source point (the beehive), two buffer zones were then considered (Figure 8):

- the first (orange line; width of the buffer radius = 1 km) was meant to localize all the beekeepers’ farms directly involved in fire episodes for the period 2007–2017;
- The second (yellow line; width of the buffer radius = 7 km) was meant to figure out the areas indirectly involved by the effects of the mentioned fire episodes.

There are 81 mapped farms, located as follows:

- Thirty-four farms in the Vesuvian area, and set at a distance of less than 1 km from the areas directly involved in fire episodes;
- Six farms within the boundaries of the Municipality of Palma Campania, and set at a distance of less than 1 km from the areas directly involved in fire episodes;
- Forty-one farms comprised between 1 km and 7 km from the areas directly involved in fire episodes. In particular, four farms are outside the Vesuvius’ red zone (two in the Municipality of Pomigliano d’Arco, and two in the Municipality of Nola).

The results of the analysis of the data highlighted that, since all the farms are set within the radius of the external buffer, it is highly likely that all the bees have been affected by the disaster episodes that occurred in the period of study.

**Figure 9:**
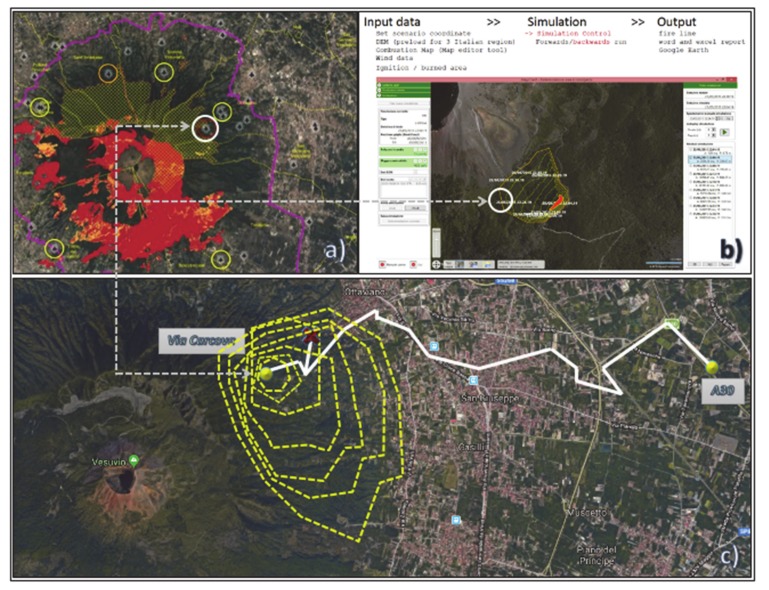
(**a**) mapping of the beekeepers’ farms in Mount Vesuvius’ red zone; (**b**) example of the TIGER simulation tool; (**c**) evaluation of the OPERA for a beekeeper farm.

**Figure 10:**
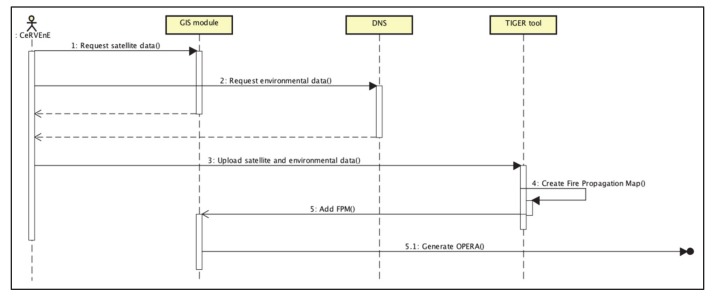
Sequence Diagram of the integrated ICT system.

Figure 9 reports the main working steps of the introduced ICT systems towards the definition of the OPERA for the case of a beekeeper farm set in via Carcova (starting junction/node) on Mount Vesuvius’ northeast slope, up to the safe zone located at the nearest entrance of the A30 highway (ending junction/node). The map in Figure 9a was obtained by means of a GIS software for the purposes of the FRAC program. It shows the starting node within a white circle, along with the other beekeepers’ sites standing on the territory under consideration, characterized by different fire risk areas (from yellow to red, in increasing danger level). In Figure 9b, the TIGER simulation tool is run, considering a fire source quite close to the beekeeper’s site. In this case, the spreading of the fire front is calculated setting up a 5-min distance between two consecutive fire perimeters. In Figure 9c, both the simulation- and the route optimization-focused aspects have been combined as a two-layers representation on a Google Maps © chart: on the one side, the dashed yellow perimeters refer to the spreading of the fire front. In this case the simulation was run considering a 10-min distance between two consecutive fire perimeters. On the other side, the segmented white line was obtained by implementing the model from the Equation (8) that made it possible to identify the single junction-to-junction edges, then manually connected so as to draw the OPERA for the case study. The figure also shows the red cross which points out that that particular node, according to the simulation, would have already been reached by the fire by the time the token was supposed to get there, therefore it was necessary to calculate another path among all the available ones. The OPERA was eventually calculated for the case analyzed, and resulted to be equal to 16, i.e., the minimum path to get from the beekeeper farm in via Carcova to the A30 highway with a single vehicle to escape fire, comprising 16 nodes/junctions. Eventually, the UML sequence diagram of the ICT system is depicted in Figure 10 [56].

## 7. Discussion and Concluding Remarks

The conceptual framework of the DSN-based system introduced in the present work represents a trustworthy data source for multi-risk models as it contributes in the different phases of a disaster management cycle, as has been described in Figure 1 [57].

With reference to the Planning/Pre-Disaster phase, its main contribution lies in helping the organization of effective actions for disaster mitigation, thanks to the possibility to previsualize escape routes so that timely and effective safety protocols can be figured out for each different animal species living in the concerned area. For what concerns the Disaster phase, the development of an advanced sensor network as early-warning system is on the other hand the key for the detection of precursory signals, which serves in short-term hazard prediction and in the activation of early warnings. It is as well called to play, in our case, a guiding role in fire-related emergency situations by offering real-time synoptic views on the affected areas, thus providing the necessary damage-related information to increase knowledge about loss distribution in the entire involved ecosystem. The Recovery/Post-Disaster phase relies eventually on the low-cost monitoring and evaluation of the recovery and reconstruction actions offered by remote sensing imagery. The monitoring is carried out through the analysis of a time series of satellite imagery that can be used e.g., to track the vegetation recovery after a wildfire.

That said, the development of such integrated system turns out as critical, since the fire risk (and especially arsons risk) has considerably increased in recent years in the whole Mount Vesuvius’ surrounding area, which features a unique combination of both animal and anthropic elements within a very delicate natural ecosystem. Actually, the intent to design and test the system on a narrow area (the mentioned “red zone”) to verify its viability also depends on another important feature of the FRAC Program, that is, the active involvement of a number of organizations (State Forestry Corps, Local Health Trusts, breeders’ associations, up to the very Regional Government) that need to strictly interface with each other—and, in case of fire, in a very short time—in order to build up a solid and efficient system of surveillance planning, risk analysis, and data gathering. This means therefore to call into account and address disaster diplomacy-related issues as well [58]. As consequences of disasters are determined by a nuanced balance between vulnerability and resilience, figuring out (and of course, putting into practice) safety protocols for animals under risk of fire implies namely: understanding of ecosystem weaknesses (mitigation); fostering relationships between different actors (planning/preparation); engaging them in creative and cooperative ways (response); handling the transition from immediate relief toward longer-term development aid (recovery).

Under such a perspective, the implementation of a specific integrated system to support the mandate of the FRAC Program is supposed to boost an improvement for the CeRVEnE’s activities, pursuant of the innovation perspectives coming with the effectual application of the disaster relief supply chain quality management principles. To such a purpose, the next steps to undertake will be particularly focused on the active involvement of the mentioned organizations, as future users of the ICT system here described, in order to funnel their vision and experience into the actual realization activities, thus enhancing robust design-based performances and routing protocols [59].
